# Perioperative outcomes of uniportal versus three-port video-assisted thoracoscopic surgery in lung cancer patients aged ≥ 75 years old: a cohort study

**DOI:** 10.1186/s12893-024-02320-7

**Published:** 2024-01-23

**Authors:** Xingqi Mi, Zhangyi Dai, Chengwu Liu, Jiandong Mei, Yunke Zhu, Lunxu Liu, Qiang Pu

**Affiliations:** Department of Thoracic Surgery, West China Hospital, Sichuan University, No. 37, Guoxue Alley, Chengdu, 610041 Sichuan China

**Keywords:** Lung cancer, Video-assisted thoracoscop ic surgery (VATS), Perioperative outcomes, The elderly, Propensity score matching (PSM)

## Abstract

**Background:**

Increasing attention has been raised on the surgical option for lung cancer patients aged ≥75 years, however, few studies have focused on whether uniportal video-assisted thoracoscopic surgery (VATS) is safe and feasible for these patients. This study aimed to evaluate short-term results of uniportal versus three-port VATS for the treatment of lung cancer patients aged ≥75 years.

**Methods:**

We retrospectively evaluated 582 lung cancer patients (≥75 years) who underwent uniportal or three-port VATS from August 2007 to August 2021 based on the Western China Lung Cancer Database. The baseline and perioperative outcomes between uniportal and three-port VATS were compared in the whole cohort (WC) and the patients undergoing lobectomy (lobectomy cohort, LC) respectively. Propensity score matching (PSM) was used to minimize confounding bias between the uniportal and three-port cohorts in WC and LC.

**Results:**

Intraoperative blood loss was significantly less in the uniportal than three-port LC (50 mL vs. 83 mL, *P* = 0.007) before PSM and relatively less in the uniportal than three-port LC (50 mL vs. 83 mL, *P* = 0.05) after PSM. Significantly more lymph nodes harvested (13 vs. 9, *P* = 0.007) were found in the uniportal than three-port LC after PSM. In addition, in WC and LC, there were no significant differences between uniportal and three-port cohorts in terms of operation time, the rate of conversion to thoracotomy during surgery, nodal treatments (dissection or sampling or not), the overall number of lymph node stations dissected, postoperative complications, volume and duration of postoperative thoracic drainage, hospital stay after operation and hospitalization expenses before and after PSM (*P* > 0.05).

**Conclusions:**

There were no significant differences in short-term outcomes between uniportal and three-port VATS for lung cancer patients (≥75 years), except relatively less intraoperative blood loss (*P <* 0.05 before PSM and *P =* 0.05 after PSM) and significantly more lymph nodes harvested (*P <* 0.05 after PSM) were found in uniportal LC. It is reasonable to indicate that uniportal VATS is a safe, feasible and effective operation procedure for lung cancer patients aged ≥75 years.

## Introduction

Recent studies have reported that lung cancer maintained the first leading cause of cancer deaths in China and worldwide [1, 2]. Surgical resection is recognized as the primary treatment method for lung cancer patients in early stage [3]. Video-assisted thoracoscopic surgery (VATS) has been considered superior to thoracotomy for less postoperative mortality and greater long-term survival [4]. The incision design of VATS has evolved from multiport into uniport due to minimizing surgical trauma. Results from several studies demonstrated the safety and effectiveness of uniportal VATS with better postoperative recovery and quality of life compared with three-port VATS [5, 6].

Similarly to younger patients, surgical intervention is also accepted as a major treatment option for the elderly with early lung cancer [7]. Meanwhile, comparing with lobectomy, sublobar resection can be also adequate for older lung cancer patients in early stage [8]. Wheras, it has been demonstrated age was a significant risk factor in surgical resection for lung cancer [9]. As the proportion of elderly patients with lung cancer has grown, older patients aged ≥75 years accounted for 25% [10]. Lung cancer patients with higher age are related to lower compliance for treatment, especially for those aged ≥75 years [11]. Meanwhile, comorbidities occurring in lung cancer patients aged ≥75 years might have impact on operative morbidity [7]. The therapy for lung cancer patients aged ≥75 years was one of the essential components of thoracic oncology.

So far, a few studies have been carried out on lung cancer patients (≥75 years) undergoing pulmonary resection, observing operative results and survival [7, 12, 13]. However, few studies have focused on the comparison of short-term outcomes between uniportal and three-port VATS for lung cancer patients aged ≥75 years. To evaluate the safety, feasibility and effectiveness of uniportal VATS for elderly lung cancer patients, we retrospectively compared perioperative outcomes of lung cancer patients (≥75 years) who underwent uniportal or three-port VATS in our hospital. We present the following article in accordance with the STROBE reporting checklist.

## Methods

### Data source

The study has been granted ethics approval by the Institutional Ethics Committee of West China Hospital, Sichuan University [No. 2022–1450]. Data used in the study was from the Western China Lung Cancer Database (WCLCD), a prospectively maintained database, which documented all the clinical records of patients with lung cancer undergoing surgical treatment at the Department of Thoracic Surgery, West China Hospital of Sichuan University since September 2005.

### Study design

**Fig. 1:**
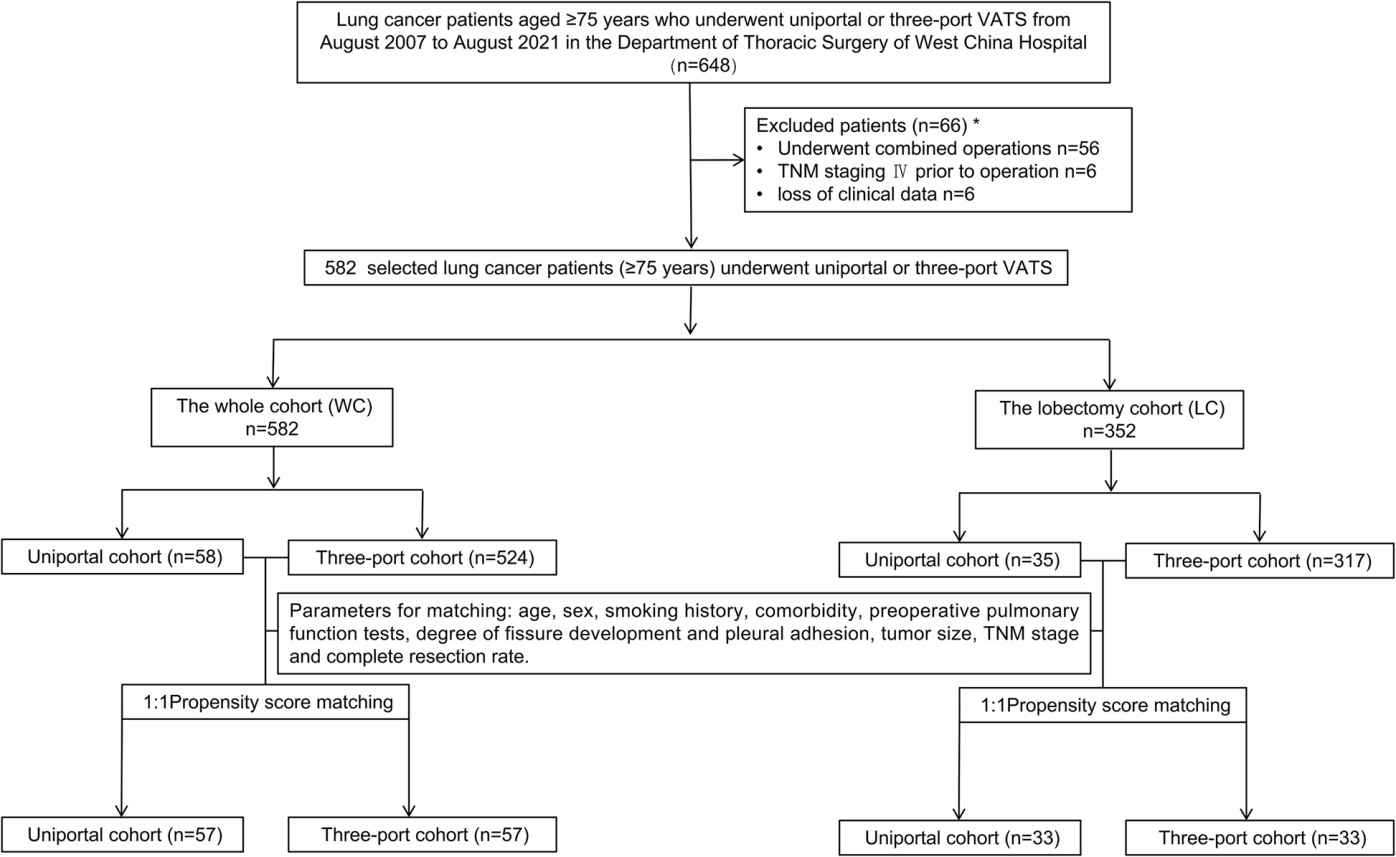
Flow diagram for eligible patients enrollment. *: The number of each exclusion standard cannot be added up to the total number of excluded patients because one patient may match multiple criteria. VATS: Video-assisted thoracoscopic surgery. TNM: Tumor-node-metastasis

Clinical records of 648 lung cancer patients (≥75 years) who had undergone uniportal or three-port VATS from August 2007 to August 2021 in the Department of Thoracic Surgery of West China Hospital were retrospectively evaluated, based on the WCLCD. Patients were extracted using the following inclusion criteria: (i) age ≥ 75 years, (ii) pathologically diagnosed lung cancer, (iii) underwent uniportal or three-port VATS lobectomy, segmentectomy or wedge resection. Patients were eliminated by the following exclusion criteria: (i) underwent combined operations other than lobectomy, segmentectomy or wedge resection, (ii) tumor-node-metastasis (TNM) staging IV prior to operation, (iii) loss of clinical data. Finally, 582 patients were identified by selection for this retrospective research. They were divided into a uniportal cohort (*n* = 58) and a three-port cohort (*n* = 524) on the basis of the operative approach used (Fig. 1). There were not enough patients undergoing segmentectomy (uniportal cohort: *n* = 11, three-port cohort: *n* = 108) and wedge resection (uniportal cohort: *n* = 12, three-port cohort: *n* = 99) for individual analysis; thereby the study analyzed the clinical records between uniportal and three-port VATS in the whole cohort (WC) and the patients undergoing lobectomy (lobectomy cohort, LC). To minimize potential confounding bias, propensity score matching (PSM) with the rate of 1:1 was performed in WC and LC. Propensity scores were calculated based on several variables including age, sex, smoking history, comorbidity, preoperative pulmonary function tests, degree of fissure development and pleural adhesion, tumor size, TNM stage and complete resection rate. We used the method of nearest neighbor matching to perform PSM and the matching tolerance was 0.02. The number of the patients enrolled in each cohort after PSM were shown in Fig. 1.

### Clinical records

The clinical records of the patients consisted of general characteristics and perioperative outcomes. General characteristics included age, sex, smoking history, comorbidity, preoperative pulmonary function tests, degree of fissure development and pleural adhesion, tumor size, TNM stage, surgical methods and complete resection rate. Perioperative outcomes involved intraoperative blood loss, operation time, the rate of conversion to thoracotomy during surgery, nodal treatments (dissection or sampling or no treatment), the overall number of lymph nodes and stations dissected, postoperative complications, volume and duration of postoperative thoracic drainage, hospital stay after operation and hospitalization expenses. TNM staging was classified according to the eighth edition of the American Joint Committee on Cancer (AJCC) criterion. Persistent pulmonary leakage was considered as consecutive air leakage lasting for more than 5 days after the operation and the explanation of persistent thoracic drainage was persistent post-operation drainage continuing for more than 6 days.

### Surgical technique

All patients underwent standard pulmonary resection and the approaches including lobectomy, segmentectomy or wedge resection were determined by age, respiratory function, tumor characteristics and comorbidities in each patient. The selection of uniportal or three-port VATS in each patient was decided by the surgeon in charge and both uniportal and three-port VATS were performed by the same surgeons. The patient was placed in a lateral position and the standing side of the surgeon was anterior to the patient. As described in our published articles [14], the positions of surgical incisions in three-port VATS were listed as follow. The thoracoport (1 cm) was made in the seventh intercostal space at the midaxillary line. The main operation port with a length of 3–4 cm was placed at the anterior axillary line in the third intercostal space for operation of upper and middle lobe and in the fourth intercostal space for operation of lower lobe. The assistant port with the incision of 2 cm was in the 9th intercostal space between the posterior axillary line and subscapular line. The single port incision (4 cm) of uniportal VATS was generally placed at the fourth intercostal space on the midaxillary line and a wound protector was placed around the incision for stretching the port. Both uniportal and three-port VATS were performed using the high-definition 30° 10 mm thoracoscope. After the resection of the pulmonary nodule, nodal dissection or sampling was carried out when necessary such as swollen lymph nodes. The tumor and the harvested lymph nodes were sent for cryosection during the surgery. Once the cryosection showed the primary cancer, the hilar and mediastinal lymph node dissection were performed. When the pathological diagnosis was negative for lymph nodes, systematic lymph node sampling was sometimes performed instead of systematic nodal dissection.

### Statistical analysis

This study retrospectively compared baseline characteristics and perioperative outcomes between uniportal and three-port cohorts in WC and LC whose age is ≥75 years. Both the data before and after PSM were analyzed in the study. Kolmogorov-Smirnov normality tests were conducted for quantitative data. Quantitative data in accordance with normal distribution were described as mean ± standard deviation (SD) and analyzed via the Student’s t-test. Non-normally distributed variables were summarized as the median and inter-quartile range (P25, P75) and examined by the Mann-Whitney test. Pearson’s Chi-square test or Fisher’s exact test was applied to assess differences in categorical data when appropriate and which were expressed as frequency and percentage (%). *P*-values < 0.05 on two sides were regarded as a statistically significant difference in statistical analyses. All data were statistically processed with SPSS Version 21.0 for Windows (IBM).

## Results

### Study cohort

Totally, 582 lung cancer patients (≥75 years) who underwent uniportal or three-port VATS from August 2007 to August 2021 in our hospital were enrolled in the study. Before PSM, in WC, 58 patients underwent uniportal VATS and 524 patients underwent three-port VATS and 35 patients underwent uniportal VATS and 317 patients underwent three-port VATS in LC (Fig. 1). After PSM, 57 patients underwent uniportal and three-port VATS respectively in WC and 33 patients underwent uniportal and three-port VATS respectively in LC (Fig. 1).

### General characteristics in WC and LC

The detailed general characteristics in WC and LC before and after PSM were respectively set out in Tables 1 and 2. In the unmatched cohorts, there were significantly more male patients in the three-port than in the uniportal cohort in LC (64.04% vs. 45.71%, *P* = 0.03) and there was no significant difference between the two cohorts in gender in WC. After PSM, no significant difference was shown between the uniportal and three-port cohorts in gender in WC an LC. The proportion of each operation procedure between uniportal and three-port cohorts in WC were nearly similar (before PSM, lobectomy 60.30% vs. 60.50%, segmentectomy 19.00% vs. 20.60%, wedge resection 20.70% vs. 19.00%, *P* = 0.92; after PSM, lobectomy 61.40% vs. 45.60%, segmentectomy 19.30% vs. 33.30%, wedge resection 19.30% vs. 21.10%, *P* = 0.17). In WC and LC before and after PSM, no statistically significant differences were presented between uniportal and three-port cohorts in terms of age, smoking history, comorbidity, predicted forced expiratory volume in 1 second (FEV1%), predicted forced vital capacity (FVC%), size of tumor, degree of fissure development and pleural adhesion, TNM stage and complete resection rate.

**Table 1:** General characteristics in whole cohort before and after propensity score matching

Clinical records	Before PSM				After PSM			
	Uniportal cohort (*n* = 58)	Three-port cohort (*n* = 524)	Z/X^2^-value	*p*-value	Uniportal cohort (*n* = 57)	Three-port cohort (*n* = 57)	Z/X^2^-value	*p*-value
Age (years)	78(76, 79)	77(76, 79)	—1.29	0.20	78(76, 79)	78(76, 79)	—0.02	0.98
Sex			0.03	0.87			0.33	0.57
Male	33(56.90%)	304(58.00%)			32(56.10%)	35(61.40%)		
Female	25(43.10%)	220(42.00%)			25(43.90%)	22(38.60%)		
Smoking history			1.42	0.23			1.72	0.20
Yes	26(44.80%)	193(36.80%)			25(43.90%)	32(56.10%)		
No	32(55.20%)	331(63.20%)			32(56.10%)	25(43.90%)		
Comorbidity	42(72.40%)	387(73.90%)	0.06	0.81	41(71.90%)	40(70.20%)	0.04	0.84
Diabetes mellitus	12(20.70%)	91(17.40%)	0.40	0.53	11(19.30%)	13(22.80%)	0.21	0.65
Hypertension	29(50.00%)	242(46.20%)	0.31	0.58	28(49.10%)	28(49.10%)	< 0.0001	> 0.99
COPD	3(5.20%)	57(10.90%)	1.84	0.18	3(5.30%)	1(1.80%)	0.26	0.61
CHD	8(13.80%)	67(12.80%)	0.05	0.83	8(14.00%)	7(12.30%)	0.08	0.78
History of other cancer	9(15.50%)	55(10.50%)	1.35	0.25	8(14.00%)	5(8.80%)	0.78	0.38
FEV1%	100.25(88.65, 112.68)	103.67(90.30, 115.13)	—1.22	0.22	100.00(88.50, 113.05)	98.00(86。35, 106.05)	—0.56	0.57
FVC%	106.20(90.88, 115.25)	109.57(97.55, 118.88)	—1.50	0.13	105.80(90.75, 115.70)	103.20(90.80, 109.94)	—0.85	0.39
Degree of fissure development			5.00	0.08			—1.18	0.08
No developemnt	1(1.70%)	9(1.70%)			1(1.80%)	0(0.00%)		
Incomplete development	27(46.60%)	323(61.60%)			27(47.40%)	19(33.33%)		
Well development	30(51.70%)	192(36.60%)			29(50.90%)	38(66.70%)		
Degree of pleural adhesion			1.35	0.25			1.90	0.17
Have adhesion	48(82.80%)	398(76.00%)			48(84.20%)	42(73.70%)		
No adhesion	10(17.20%)	126(24.00%)			9(15.80%)	15(26.30%)		
Tumor size (cm)	2.00(1.50, 2.93)	2.20(1.50, 3.20)	—0.77	0.44	2.00(1.50, 2.95)	2.00(1.55, 3.00)	—0.21	0.83
TNM stage			—1.53	0.13			—1.81	0.84
IA	42(72.40%)	338(64.50%)			41(71.90%)	42(73.70%)		
IB	8(13.80%)	64(12.20%)			8(14.00%)	8(14.00%)		
IIA	5(8.60%)	25(4.80%)			5(8.80%)	1(1.80%)		
IIB	0(0.00%)	34(6.50%)			0(0.00%)	3(5.30%)		
IIIA	2(3.40%)	50(9.50%)			2(3.50%)	3(5.30%)		
IIIB	1(1.70%)	13(2.50%)			1(1.80%)	0(0.00%)		
Operative procedure			0.16	0.92			3.51	0.17
Lobectomy	35(60.30%)	317(60.50%)			35(61.40%)	26(45.60%)		
Segmentectomy	11(19.00%)	108(20.60%)			11(19.30%)	19(33.30%)		
Pulmonary wedge resection	12(20.70%)	99(18.90%)			11(19.30%)	12(21.10%)		
Complete resection			–	> 0.99			–	–
Yes	58(100.00%)	521(99.40%)			57(100.00%)	57(100.00%)		
No	0(0.00%)	3(0.60%)			0(0.00%)	0(0.00%)		

— statistics cannot be provided for Fisher’s precision probability test applied into this variable; *PSM* Propensity score matching, *COPD* Chronic obstructive pulmonary disease, *CHD* Coronary heart disease, *FEV*1% Predicted forced expiratory volume in 1 second: *FVC*% Predicted forced vital capacity, *TNM* Tumor-node-metastasis

**Table 2:** General characteristics in lobectomy cohort before and after propensity score matching

Clinical records	Before PSM				After PSM			
	Uniportal cohort (*n* = 35)	Three-port cohort (*n* = 317)	Z/X^2^-value	*p*-value	Uniportal cohort (*n* = 33)	Three-port cohort (*n* = 33)	Z/X^2^-value	*p*-value
Age (years)	78(76, 79)	77(76, 79)	—1.00	0.32	77(76, 79)	77(76, 78)	—0.70	0.48
Sex			4.50	0.03			0.24	0.62
Male	16(45.70%)	203(64.00%)			16(48.50%)	14(42.40%)		
Female	19(54.30%)	114(36.00%)			17(51.50%)	19(57.60%)		
Smoking history			0.001	0.98			< 0.0001	> 0.99
Yes	15(42.90%)	135(42.60%)			15(45.50%)	15(45.50%)		
No	20(57.10%)	182(57.40%)			18(54.50%)	18(54.50%)		
Comorbidity	23(65.70%)	223(70.30%)	0.32	0.57	21(63.60%)	20(60.60%)	0.06	0.80
Diabetes mellitus	5(14.30%)	48(15.10%)	0.02	0.89	5(15.20%)	3(9.10%)	0.57	0.45
Hypertension	16(45.70%)	137(43.20%)	0.08	0.78	14(42.40%)	14(42.40%)	< 0.0001	> 0.99
COPD	2(5.70%)	38(12.00%)	0.69	0.41	2(6.10%)	1(3.00%)	0.35	0.56
CHD	7(20.00%)	41(12.90%)	0.80	0.37	5(15.20%)	5(15.20%)	< 0.0001	> 0.99
History of other cancer	3(8.60%)	23(7.30%)	< 0.0001	> 0.99	3(9.10%)	1(3.00%)	0.27	0.61
FEV1%	99.90(86.90, 114.40)	103.67(89.75, 111.85)	—0.40	0.69	103.67(89.80, 116.05)	103.67(90.85, 125.60)	—0.62	0.53
FVC%	105.70(91.10, 122.80)	109.57(96.85, 117.45)	—0.54	0.59	106.60(96.95, 124.10)	108.90(97.45, 126.35)	—0.41	0.68
Degree of fissure development			1.70	0.43			—0.71	0.48
No developemnt	1(2.90%)	8(2.50%)			1(3.00%)	1(3.00%)		
Incomplete development	18(51.40%)	199(62.80%)			17(51.50%)	20(60.60%)		
Well development	16(45.70%)	110(34.70%)			15(45.50%)	12(36.40%)		
Degree of pleural adhesion			0.47	0.50			1.22	0.27
Have adhesion	28(80.00%)	237(74.80%)			26(78.80%)	22(66.70%)		
No adhesion	7(20.00%)	80(25.20%)			7(21.20%)	11(33.30%)		
Tumor size (cm)	2.40(1.80, 3.20)	2.70(2.00, 3.80)	—1.41	0.16	2.40(1.80, 3.35)	2.30(1.95, 2.75)	—0.67	0.50
TNM stage			—1.45	0.15			—0.90	0.37
IA	21(60.00%)	161(50.80%)			19(57.60%)	23(69.70%)		
IB	7(20.00%)	52(16.40%)			7(21.20%)	4(12.10%)		
IIA	4(11.40%)	25(7.90%)			4(12.10%)	3(9.10%)		
IIB	0(0.00%)	30(9.50%)			0(0.00%)	1(3.00%)		
IIIA	2(5.70%)	36(11.40%)			2(6.10%)	2(6.10%)		
IIIB	1(2.90%)	13(4.10%)			1(3.00%)	0(0.00%)		
Complete resection			–	> 0.99			–	–
Yes	35(100.00%)	315(99.40%)			33(100.00%)	33(100.00%)		
No	0(0.00%)	2(0.60%)			0(0.00%)	0(0.00%)		

— statistics cannot be provided for Fisher’s precision probability test applied into this variable; *PSM* Propensity score matching, *COPD* Chronic obstructive pulmonary disease, *CHD* Coronary heart disease, *FEV*1% Predicted forced expiratory volume in 1 second, *FVC*% Predicted forced vital capacity, *TNM* Tumor-node-metastasis

### Perioperative outcomes in WC and LC

Tables 3 and 4 respectively compares perioperative outcomes between uniportal and three-port cohorts in WC and LC before and after PSM. In the unmatched cohort, what stood out in the comparison was that intraoperative blood loss was significantly less in uniportal than in three-port VATS in LC (50 mL vs. 83 mL, *P* = 0.007). Meanwhile, uniportal VATS was relevant to relatively less intraoperative blood loss compared with three-port VATS in WC (50 mL vs. 75 mL, *P* = 0.05). After PSM, intraoperative blood loss was relatively less in uniportal than in three-port VATS in LC (50 mL vs. 83 mL, *P* = 0.05) and in WC (50 mL vs. 80 mL, *P* = 0.09). After PSM, the overall number of lymph nodes dissected was significantly more in the uniportal than three-port cohort in LC (13 vs. 9, *P* = 0.007) and no significant differencs between uniportal and three-port VATS were shown in the overall number of lymph nodes dissected in WC (8 vs. 7, *P* = 0.40). In WC and LC, no significant differences between uniportal and three-port VATS were shown in nodal treatments (dissection or sampling or no treatment) and were also observed in the overall number of lymph node stations dissected before and after PSM. There were no significant differences between uniportal and three-port cohorts in WC and LC in terms of operation time, the rate of conversion to thoracotomy during surgery, volume and duration of postoperative thoracic drainage, hospital stay after operation and hospitalization expenses before and after PSM. No deaths were observed within postoperative 30 days in all patients. The postoperative complications according to the Clavien-Dindo classification in WC and LC were reported in Tables 5 and 6. There were seven patients (1.20%) with major complications (Clavien-Dindo grade 3–5) and 101 patients (17.40%) with minor complications (Clavien-Dindo grade 1–2) in WC and six patients (1.70%) with major complications (Clavien-Dindo grade 3–5) and 76 patients (21.60%) with minor complications (Clavien-Dindo grade 1–2) in LC. No significant differences between uniportal and three-port cohorts in WC and LC in postoperative complications were shown before and after PSM. After the patients being discharged, several complications observed during the follow-up time were listed in Tables 5 and 6. There were no significant differences in post-discharge complications between the two cohorts in WC and LC before and after PSM.

**Table 3:** Perioperative outcomes in the whole cohort before and after propensity score matching

Clinical records	Before PSM				After PSM			
	Uniportal cohort (*n* = 58)	Three-port cohort (*n* = 524)	Z/X^2^-value	*p*-value	Uniportal cohort (*n* = 57)	Three-port cohort (*n* = 57)	Z/X^2^-value	*p*-value
Intraoperative blood loss (mL)	50(20, 83)	75(20, 83)	—1.95	0.05	50(20, 83)	80(25, 83)	—1.68	0.09
Operation time (min)	108(84, 131)	110(76, 149)	—0.04	0.97	110(85, 132)	110(73, 133)	—0.51	0.61
Conversion to thoracotomy rate	4(6.90%)	20(3.80%)	0.60	0.44	4(7.00%)	2(3.50%)	0.18	0.68
Lymph node dissection			1.64	0.44			0.54	0.76
Nodal dissection	48(82.80%)	441(84.20%)			48(84.20%)	49(86.00%)		
Nodal sampling	7(12.10%)	41(7.80%)			7(12.30%)	5(8.80%)		
No nodal dissection	3(5.20%)	42(8.00%)			2(3.50%)	3(5.30%)		
Overall number of lymph nodes dissected	10(5, 18)^a^	9(6, 14)^b^	—1.02	0.31	8(5, 16)^c^	7(4, 13)^d^	—0.84	0.40
Overall number of lymph node stations dissected	5(3, 6)^a^	5(3, 6)^b^	—0.52	0.60	5(3, 6)^c^	5(3, 6)^d^	—0.01	> 0.99
Postoperative thoracic drainage (mL)								
Within postoperative 3 days	480(349, 733)	495(260, 805)	—0.24	0.81	500(348, 745)	520(315, 850)	—0.32	0.75
Total	655(373, 1093)	570(270, 1125)	—0.67	0.50	660(365, 1095)	560(315, 1123)	—0.39	0.70
Duration of thoracic drainage (day)	3(2, 5)	3(2, 5)	—0.71	0.48	3(2, 5)	3(2, 5)	—0.88	0.38
Postoperative length of stay (day)	6(4, 9)	6(4, 8)	—0.36	0.72	6(4, 9)	5(4, 7)	—0.57	0.57
Hospitalization expenses (¥)	54,004.42 (50,285.98, 60,978.32)	54,320.23 (46,887.35, 61,897.48)	—0.77	0.44	53,988.58 (50,281.47, 61,665.97)	56,537.34 (48,606.79, 67,027.76)	—1.30	0.20

—: statistics cannot be provided for Fisher’s precision probability test applied into this variable; *PSM* Propensity score matching; ^a^ Including 48 patients performed nodal dissection in the uniportal cohort before propensity score matching; ^b^ Including 441 patients performed nodal dissection in the three-port cohort before propensity score matching; ^c^ Including 48 patients performed nodal dissection in the uniportal cohort after propensity score matching; ^d^ Including 49 patients performed nodal dissection in the three-port cohort after propensity score matching

**Table 4:** Perioperative outcomes in the lobectomy cohort before and after propensity score matching

Clinical records	Before PSM				After PSM			
	Uniportal cohort (*n* = 35)	Three-port cohort (*n* = 317)	Z/X^2^-value	*p*-value	Uniportal cohort (*n* = 33)	Three-port cohort (*n* = 33)	Z/X^2^-value	*p*-value
Intraoperative blood loss (mL)	50(20, 83)	83(30, 83)	—2.72	0.007^e^	50(20, 83)	83(30, 83)	—1.94	0.05
Operation time (min)	125(103, 150)	120(95, 164)	—0.19	0.85	125(101, 148)	119(92, 161)	—0.70	0.48
Conversion to thoracotomy rate	4(11.40%)	19(6.00%)	0.76	0.38	4(12.10%)	2(6.10%)	0.18	0.67
Lymph node dissection			3.21	0.20			4.30	0.12
Nodal dissection	35(100.00%)	302(95.30%)			33(100.00%)	30(90.90%)		
Nodal sampling	0(0.00%)	8(2.50%)			0(0.00%)	1(3.00%)		
No nodal dissection	0(0.00%)	7(2.20%)			0(0.00%)	2(6.10%)		
Overall number of lymph nodes dissected	13(7, 19)^a^	11(7, 16)^b^	—0.97	0.33	13(10, 20)^c^	9(5, 14)^d^	—2.69	0.007^e^
Overall number of lymph node stations dissected	6(5, 7)^a^	5(4, 6)^b^	—1.53	0.13	6(5, 7)^c^	6(4, 7)^d^	—0.71	0.48
Postoperative thoracic drainage (mL)								
Within postoperative 3 days	500(350, 810)	590(340, 870)	—0.57	0.57	510(350, 855)	640(410, 875)	—0.67	0.51
Total	660(400, 1190)	740(350, 1340)	—0.36	0.72	680(405, 1215)	700(410, 1265)	—0.16	0.87
Duration of thoracic drainage (day)	3(2, 5)	3(2, 6)	—0.50	0.62	3(2, 5)	3(2, 5)	—0.14	0.89
Postoperative length of stay (day)	6(4, 10)	7(5, 10)	—1.60	0.11	6(4, 10)	6(4, 9)	—0.32	0.75
Hospitalization expenses (¥)	52,405.53 (47,672.97, 56,537.34)	56,537.34 (494,998.99, 63,900.08)	—1.83	0.07	52,405.53 (47,571.81, 57,704.24)	52,791.46 (48,122.71, 60,843.34)	—0.07	0.94

— statistics cannot be provided for Fisher’s precision probability test applied into this variable; *PSM* Propensity score matching; ^a^ Including 35 patients performed nodal dissection in the uniportal cohort before propensity score matching; ^b^ Including 302 patients performed nodal dissection in the three-port cohort before propensity score matching; ^c^ Including 33 patients performed nodal dissection in the uniportal cohort after propensity score matching; ^d^ Including 30 patients performed nodal dissection in the three-port cohort after propensity score matching; ^e^ statistically significant (*P* < 0.05)

**Table 5:** Postoperative complications in the whole cohort before and after propensity score matching

Clinical records	Before PSM				After PSM			
	Uniportal cohort (*n* = 58)	Three-port cohort (*n* = 524)	Z/X^2^-value	*p*-value	Uniportal cohort (*n* = 57)	Three-port cohort (*n* = 57)	Z/X^2^-value	*p*-value
Postoperative complications	11(19.00%)	97(18.50%)	0.007	0.93	11(19.30%)	10(17.50%)	0.06	0.81
Minor complications (Clavien-Dindo grade 1–2)								
Persistent thoracic drainage	6(10.30%)	63(12.00%)	0.14	0.71	6(10.50%)	6(10.50%)	< 0.0001	> 0.99
Persistent pulmonary leakage	3(5.20%)	38(7.30%)	0.10	0.75	3(5.30%)	2(3.50%)	< 0.0001	> 0.99
Pulmonary infection	2(3.40%)	34(6.50%)	0.39	0.53	2(3.50%)	5(8.80%)	0.61	0.44
Pneumoderm	1(1.70%)	14(2.70%)	< 0.0001	> 0.99	1(1.80%)	2(3.50%)	< 0.0001	> 0.99
Arrhythmia	1(1.70%)	5(1.00%)	< 0.0001	> 0.99	1(1.80%)	0(0.00%)	< 0.0001	> 0.99
Others	2(3.40%)	38(7.30%)	0.66	0.42	2(3.50%)	3(5.30%)	< 0.0001	> 0.99
Major complications (Clavien-Dindo grade 3–5)								
Dyspnea	0(0.00%)	5(1.00%)	< 0.0001	> 0.99	0(0.00%)	1(0.20%)	< 0.0001	> 0.99
Pulmonary infection	0(0.00%)	4(0.80%)	< 0.0001	> 0.99	0(0.00%)	0(0.00%)	–	–
Chylothorax	0(0.00%)	3(0.60%)	< 0.0001	> 0.99	0(0.00%)	0(0.00%)	–	–
Pulmonary embolism	0(0.00%)	1(0.20%)	< 0.0001	> 0.99	0(0.00%)	0(0.00%)	–	–
Post-discharge complications	3(5.20%)	22(4.20%)	< 0.0001	> 0.99	3(5.30%)	3(5.30%)	< 0.0001	> 0.99
Generalized pruritus	0(0.00%)	4(0.80%)	< 0.0001	> 0.99	0(0.00%)	2(3.50%)	0.51	0.48
Chest pain	2(3.40%)	7(1.30%)	0.46	0.50	2(3.50%)	1(1.80%)	< 0.0001	> 0.99

— statistics cannot be provided for Fisher’s precision probability test applied into this variable; *PSM* Propensity score matching

**Table 6:** Postoperative complications in the lobectomy cohort before and after propensity score matching

Clinical records	Before PSM				After PSM			
	Uniportal cohort (*n* = 35)	Three-port cohort (*n* = 317)	Z/X^2^-value	*p*-value	Uniportal cohort (*n* = 33)	Three-port cohort (*n* = 33)	Z/X^2^-value	*p*-value
Postoperative complications	6(17.10%)	76(24.00%)	0.82	0.36	5(15.20%)	4(12.10%)	< 0.0001	> 0.99
Minor complications (Clavien-Dindo grade 1–2)								
Persistent thoracic drainage	4(11.40%)	48(15.10%)	0.35	0.56	4(12.10%)	4(12.10%)	< 0.0001	> 0.99
Persistent pulmonary leakage	2(5.70%)	28(8.80%)	0.10	0.76	2(6.10%)	1(3.00%)	< 0.0001	> 0.99
Pulmonary infection	1(2.90%)	27(8.50%)	0.71	0.40	1(3.00%)	0(0.00%)	< 0.0001	> 0.99
Pneumoderm	0(0.00%)	12(3.80%)	0.46	0.50	0(0.00%)	1(3.00%)	< 0.0001	> 0.99
Arrhythmia	1(2.90%)	5(1.60%)	< 0.0001	> 0.99	0(0.00%)	0(0.00%)	–	–
Others	1(2.90%)	26(8.20%)	0.63	0.43	1(3.00%)	0(0.00%)	< 0.0001	> 0.99
Major complications (Clavien-Dindo grade 3–5)								
Dyspnea	0(0.00%)	4(1.30%)	< 0.0001	> 0.99	0(0.00%)	0(0.00%)	–	–
Pulmonary infection	0(0.00%)	2(0.60%)	< 0.0001	> 0.99	0(0.00%)	0(0.00%)	–	–
Chylothorax	0(0.00%)	2(0.60%)	< 0.0001	> 0.99	0(0.00%)	0(0.00%)	–	–
Pulmonary embolism	0(0.00%)	1(0.30%)	< 0.0001	> 0.99	0(0.00%)	0(0.00%)	–	–
Post-discharge complications	1(2.90%)	16(5.00%)	0.03	0.87	1(3.00%)	3(9.10%)	0.27	0.61
Generalized pruritus	0(0.00%)	2(0.60%)	< 0.0001	> 0.99	0(0.00%)	1(3.00%)	< 0.0001	> 0.99
Chest pain	1(2.90%)	5(1.60%)	< 0.0001	> 0.99	1(3.00%)	2(6.10%)	< 0.0001	> 0.99

— statistics cannot be provided for Fisher’s precision probability test applied into this variable; *PSM *Propensity score matching

## Discussion

Surgery is generally accepted as a major treatment option for the patients with early-stage lung cancer [3], containing older people [15]. The patients aged ≥75 years have been eliminated in most clinical studies on surgical treatments for lung cancer when selecting patients. However, with the advent of the aging age, more lung cancer patients (≥75 years) in all stages appeared to be poorer adherence to treatment than younger patients and age seemed to be the most important factor on therapy decision for lung cancer patients [11]. Advanced grade of Adult Comorbidity Evaluation-27 index that was used to assess preoperative comorbidities has proven a significantly poor prognostic factor for lung cancer patients (≥75 years) undergoing surgery [12]. Recently, increasing attention has been raised on the surgical option for lung cancer patients aged ≥75 years. This study was conducted to compare perioperative outcomes between uniportal and three-port VATS for lung cancer patients aged ≥75 years. The study found no significant differences between uniportal and three-port VATS in perioperative outcomes in WC and LC, except uniportal LC was associated with relatively lower intraoperative blood loss(*P <* 0.05 before PSM and *P =* 0.05 after PSM) and significantly more lymph nodes harvested (*P <* 0.05 after PSM).

With the purpose of minimal incision trauma, uniportal VATS has been introduced in the application of VATS recently [16]. Previous research has shown uniportal VATS lobectomy was superior with regard to significantly less intraoperative blood loss for the patients whose mean age ≤ 65 years [5, 17]. In this study for the elderly aged ≥75 years, intraoperative blood loss was significantly lower in uniportal than three-port VATS obectomy before PSM (*P* < 0.05) and relatively lower in uniportal than three-port VATS obectomy before PSM (*P* = 0.05). There are some mainly possible reasons to explain these results as follows. First, the single port made in the intercostal space would decrease the injury of the muscle and vessels around the incision to reduce blood loss. Second, using a soft incision protector can lower intraoperative bleeding from the incision. Finally, the insertion of all instruments and the camera through one port can provide a direct view that is more similar to that in thoracotomy, with the advantage of exact resection and avoidance of accidental damage. These results might suggest the safety of uniportal VATS in reducing bleeding during the surgery and its applicability in treating older lung cancer patients aged ≥75 years.

The rate of postoperative complications is also an important aspect in evaluating the safety of surgery, especially in the elderly. Dai et al. [18] carried out a prospective study finding that postoperative complication rates were similar between uniportal and three-port VATS lobectomy. A retrospective study conducted by Ji et al. [19] showed there was no significant difference in postoperative complication rate between uniportal and three-port VATS lobectomy and anatomic segmentectomy. In detail, the mean age of the enrolled patients in the two previous studies above were ≤ 60 and ≤ 65 years respectively. For lung cancer patients (≥75 years), the present study showed rates of postoperative total complications or each postoperative complication listed in the Tables 5 and 6 did not differ significantly between uniportal and three-port VATS in WC and LC before and after PSM. In this study, the recorded comorbidities of the patients were mainly about diabetes mellitus and cardiopulmonary function such as hypertension, chronic obstructive pulmonary disease and coronary heart disease. Moreover, the listed postoperative complications were mostly about persistent thoracic drainage, persistent pulmonary leakage, pulmonary infection, pneumoderm and chylothorax which were related to the surgery itself. Also, the post-discharge complications observed during the follow-up time were including generalized pruritus and chest pain which were not correlated to preoperative comorbidities. In this way, there was no specific connection between comorbidities and post-operation complications. These data showed uniportal VATS would not raise the incidence of postoperative complications although cardiopulmonary reserve and physical function are generally declining with aging, which indicated the safety of uniportal VATS for the elderly as well.

Mediastinal lymph node dissection plays a significant role in staging accuracy and long-term survival. Theoretically, nodal dissection by uniportal VATS would be limited due to instrument interference caused by the insertion of all instruments through the same port. Recently, two studies reported by Shen et al. [20] and Liu et al. [21] have demonstrated the total number of lymph nodes dissected via uniportal VATS was similar to that via three-port VATS in lobectomy. Meanwhile, Liu et al. [21] also found no significant difference in the stations of lymph nodes dissected between uniportal and three-port VATS lobectomy. In this study for lung cancer patients aged ≥75 years, the overall number of lymph nodes dissected was significantly more in the uniportal than three-port cohort in LC and no significant differenc between uniportal and three-port VATS was shown in the overall number of lymph nodes dissected in WC after PSM. Moreover,no significant differences were found in the stations of lymph nodes dissected between uniportal and three-port VATS in WC and LC before and after PSM. These data demonstrated uniportal VATS could reach comparable outcomes of nodal dissection compared to three-port VATS, which also provided support to the feasibility of uniportal VATS for lung cancer patients aged ≥75 years. A possible reason for these data might be that suitable placement of the instruments and the camera during uniportal VATS could reduce instrument interference and present an exposed view to remove the target lymph node through the limited space of the single port.

Shortened postoperative length of hospital stay is an important index presenting accelerated postoperative recovery. The data in this study for lung cancer patients (≥75 years) showed that postoperative hospital duration was similar between uniportal and three-port VATS in WC and LC. However, the yields in two previous studies which both analyzed lung cancer patients aged ≤75 years showed postoperative hospital stay was shorter in uniportal VATS. The two previous studies are as follows. Xu et al. [17] conducted a prospective study finding a significantly shorter postoperative admission stay in uniportal VATS lobectomy. In another study published by Lee et al. [22], postoperative length of stay was significantly shorter in uniportal VATS segmentectomy. The advantage of uniportal VATS in reducing postoperative hospital stay might not be revealed when treating older lung cancer patients, probably because the rise in comorbidities and the decline in functional condition as aging would delay postoperative recovery. In general, these results indicated that uniportal VATS was a effective surgery that would not lengthen postoperative hospital duration to slow postoperative recovery for lung cancer patients aged ≥75 years.

Postoperative pain relief would also present accelerated postoperative recovery, benefiting facilitating coughing and expectorating. Uniportal VATS would cause lower postoperative pain for lung cancer patients [5], as well as older patients potentially. The main explanation for this consideration may be that the single port design minimizes the damage to nerve and muscle around the incision, and using a soft incision protector reduces repeating press and extrusion due to the insertion of all instruments through the same port, relieving postoperative pain. However, as aging, poorer pain tolerance and more postoperative analgesia may obscure the advantages of uniportal VATS in relieving postoperative pain for older patients. Postoperative pain control in older lung cancer patients undergoing uniportal VATS is an essential issue for future research.

This study has several limitations as follow. First, this study was a retrospective study potentially resulting in analysis and selection bias, although strict inclusion and exclusion criteria were applied. Second, the size of this study were relatively small probably because lung cancer patients (≥75 years) are related to poorer adherence to treatment. Third, this study crossed a relatively longer time span, which meant that there might be a gap concerning surgical proficiency from the same surgeons between early and present days. In this way, the differences of intraoperative blood loss between early days surgery and present surgery might exist. The clinical relevance of intraoperative blood loss in this study may be slightly limited and is still need further confirmation. Thus, a multi-institution, randomized, controlled trial with larger patients and univariated and multi-variated analysis for uniportal VATS are suggested in future research.

## Conclusions

In this study, the results have shown no significant differences in perioperative outcomes between uniportal and three-port VATS for lung cancer patients aged ≥75 years, except uniportal VATS lobectomy was associated with relatively less intraoperative blood loss and significantly more lymph nodes harvested. Overall, these findings indicate that uniportal VATS is a safe, feasible and effective surgical option achieving adequate results, compared with three-port VATS, for the treatment of lung cancer patients aged ≥75 years.

## Data Availability

The datasets used and analyzed during the current study are available from the corresponding author on reasonable request.

## Abbreviations

VATS: Video-assisted thoracoscopic surgery
WCLCD: Western China Lung Cancer Database
TNM: Tumor-node-metastasis
WC: Whole cohort
LC: Lobectomy cohort
PSM: Propensity score matching
AJCC: American Joint Committee on Cancer
SD: Standard deviation
